# Effect modification of *FADS2* polymorphisms on the association between breastfeeding and intelligence: results from a collaborative meta-analysis

**DOI:** 10.1093/ije/dyy273

**Published:** 2018-12-11

**Authors:** Fernando Pires Hartwig, Neil Martin Davies, Bernardo Lessa Horta, Tarunveer S Ahluwalia, Hans Bisgaard, Klaus Bønnelykke, Avshalom Caspi, Terrie E Moffitt, Richie Poulton, Ayesha Sajjad, Henning W Tiemeier, Albert Dalmau-Bueno, Mònica Guxens, Mariona Bustamante, Loreto Santa-Marina, Nadine Parker, Tomáš Paus, Zdenka Pausova, Lotte Lauritzen, Theresia M Schnurr, Kim F Michaelsen, Torben Hansen, Wendy Oddy, Craig E Pennell, Nicole M Warrington, George Davey Smith, Cesar Gomes Victora

**Affiliations:** 1 Postgraduate Program in Epidemiology, Federal University of Pelotas, Pelotas, Brazil; 2 Medical Research Council Integrative Epidemiology Unit, University of Bristol, Bristol, UK; 3 Population Health Sciences, University of Bristol, Bristol, UK; 4 Copenhagen Prospective Studies on Asthma in Childhood, Herlev and Gentofte Hospital, University of Copenhagen, Copenhagen, Denmark; 5 Department of Psychology and Neuroscience, Duke University, Durham, NC, USA; 6 Institute of Psychiatry, Psychology, and Neuroscience, King’s College London, London, UK; 7 Department of Psychology, University of Otago, Dunedin, New Zealand; 8 Department of Epidemiology; 9 Department of Child and Adolescent Psychiatry/Psychology, Erasmus University Medical Centre, Rotterdam, The Netherlands; 10 ISGlobal, Centre for Research in Environmental Epidemiology (CREAL), Barcelona, Spain; 11 Department of Health and Life Sciences, Universitat Pompeu Fabra (UPF), Barcelona, Spain; 12 CIBER Epidemiología y Salud Pública (CIBERESP), Madrid, Spain; 13 Centre for Genomic Regulation (CRG), Barcelona Institute of Science and Technology, Barcelona, Spain; 14 Basque Country Health Department, BIODONOSTIA Health Research Institute, San Sebastian, Spain; 15 Public Health Division of Gipuzkoa, BIODONOSTIA Health Research Institute, San Sebastian, Spain; 16 Bloorview Research Institute, Holland Bloorview Kids Rehabilitation Hospital, Toronto, ON, Canada; 17 Institute of Medical Science; 18 Departments of Psychiatry and Psychology, University of Toronto, Toronto, ON, Canada; 19 Hospital for Sick Children Research Institute, Peter Gilgan Centre for Research and Learning, Toronto, ON, Canada; 20 Department of Nutritional Sciences; 21 Department of Physiology, University of Toronto, Toronto, ON, Canada; 22 Department of Nutrition, Exercise and Sports, University of Copenhagen, Copenhagen, Denmark; 23 Novo Nordisk Foundation Centre for Basic Metabolic Research, Section of Metabolic Genetics, University of Copenhagen, Copenhagen, Denmark; 24 Menzies Institute for Medical Research, University of Tasmania, Hobart, Australia; 25 School of Women’s and Infants’ Health, University of Western Australia, Perth, WA, Australia and; 26 University of Queensland Diamantina Institute, Translational Research Institute, Brisbane, QLD, Australia

**Keywords:** Breastfeeding, intelligence, FADS2, fatty acids, effect modification, meta-analysis

## Abstract

**Background:**

Accumulating evidence suggests that breastfeeding benefits children’s intelligence, possibly due to long-chain polyunsaturated fatty acids (LC-PUFAs) present in breast milk. Under a nutritional adequacy hypothesis, an interaction between breastfeeding and genetic variants associated with endogenous LC-PUFAs synthesis might be expected. However, the literature on this topic is controversial.

**Methods:**

We investigated this gene × environment interaction through a collaborative effort. The primary analysis involved >12 000 individuals and used ever breastfeeding, *FADS2* polymorphisms rs174575 and rs1535 coded assuming a recessive effect of the G allele, and intelligence quotient (IQ) in Z scores.

**Results:**

There was no strong evidence of interaction, with pooled covariate-adjusted interaction coefficients (i.e. difference between genetic groups of the difference in IQ Z scores comparing ever with never breastfed individuals) of 0.12[(95% confidence interval (CI): −0.19; 0.43] and 0.06 (95% CI: −0.16; 0.27) for the rs174575 and rs1535 variants, respectively. Secondary analyses corroborated these results. In studies with ≥5.85 and <5.85 months of breastfeeding duration, pooled estimates for the rs174575 variant were 0.50 (95% CI: −0.06; 1.06) and 0.14 (95% CI: −0.10; 0.38), respectively, and 0.27 (95% CI: −0.28; 0.82) and −0.01 (95% CI: −0.19; 0.16) for the rs1535 variant.

**Conclusions:**

Our findings did not support an interaction between ever breastfeeding and *FADS2* polymorphisms. However, subgroup analysis suggested that breastfeeding may supply LC-PUFAs requirements for cognitive development if breastfeeding lasts for some (currently unknown) time. Future studies in large individual-level datasets would allow properly powered subgroup analyses and further improve our understanding on the breastfeeding × *FADS2* interaction.

**Key Messages**

- Breastfeeding is considered to improve children’s intelligence, possibly due to long-chain polyunsaturated fatty acids (LC-PUFAs).
- The literature on the interaction between breastfeeding and variants in the *FADS2* on intelligence quotient (IQ) is controversial.
- Our *de novo* collaborative meta-analysis did not find support for this interaction when comparing ever vs never breastfed individuals.
- Subgroup analyses, although underpowered, were compatible with a role of breastfeeding duration in this interaction. This finding requires replication.

## Introduction

Breastfeeding has well-established short-term benefits on children’s health. There is also accumulating evidence that breastfeeding may benefit cognitive development.^1^ A recent meta-analysis of observational studies reported that breastfed subjects scored higher on intelligence quotient (IQ) tests [mean difference 3.4 [95% confidence interval (CI): 2.3; 4.6]} than non-breastfed subjects.^2^ Although issues such as residual confounding^3^ and publication bias^4^ could have affected this estimate, randomized controlled trials of breastfeeding promotion reported benefits in motor development in the first year of life^5^ and in IQ at 6.5 years of age.^6^ Additional studies corroborate the notion that breastfeeding has a causal effect on IQ. These include comparisons between cohorts with different confounding structures,^7^ and comparisons between mothers who tried, but could not breastfeed their child and mothers who had formula feeding as their first choice.^8^

One of the possible biological mechanisms underlying the effect of breastfeeding on IQ is through long-chain polyunsaturated fatty acids (LC-PUFAs), such as docosahexaenoic acid (DHA). Meta-analyses of randomized controlled trials of supplementation of DHA and other LC-PUFAs in infants reported improved cognitive development^9^ and visual acuity.^10^ DHA is an important component of the membrane of brain cells and retina cells.^11^^,^^12^ Studies in animal models and humans suggest that adequate levels of DHA are important for cognitive development through several processes, such as biogenesis and fluidity of cellular membranes, neurogenesis, neurotransmission and protection against oxidative stress.^12^^,^^13^

**Figure 1.**
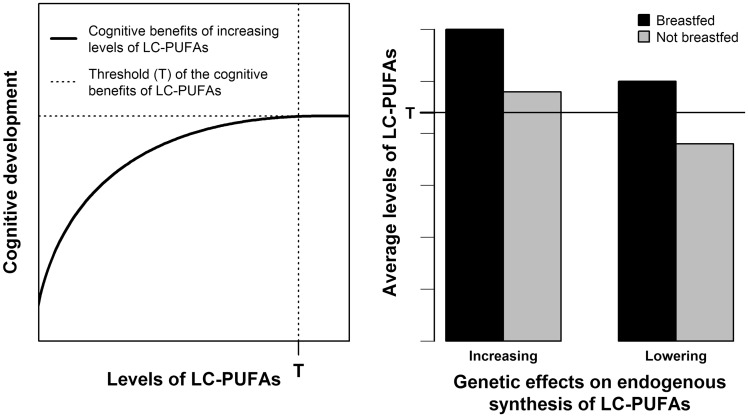
Illustration of the nutritional adequacy hypothesis involving breastfeeding, LC-PUFAs levels and associated genotypes, and cognitive development. Left panel: the benefits of increasing LC-PUFAs on cognitive development are assumed to exist only until a given level of LC-PUFAs (marked by T). Further increasing LC-PUFAs above T brings no further cognitive benefits. Right panel: breastfeeding is assumed to provide LC-PUFA levels above T regardless of genetic predisposition to higher or lower endogenous synthesis of LC-PUFAs. Non-breastfed individuals are assumed to need such genetic load of higher endogenous synthesis to achieve T. LC-PUFAs: long-chain polyunsaturated fatty acids.

The role of LC-PUFAs in the association between breastfeeding and IQ can be investigated through a gene × environment (G × E) interaction analysis. Here, we framed this G × E interaction as a nutritional adequacy hypothesis. A brief and general definition of nutritional adequacy is that, once an individual’s nutritional requirement is met, further intake of the given nutrient yields no additional benefit.^14–16^ This concept is important when defining dietary recommendations to improve nutrition and its downstream consequences, such as disease prevention.^15^^,^^16^ In the case of the present study, our nutritional adequacy hypothesis (Figure 1) postulates that there is an upper limit for the benefits of increasing DHA levels (Figure 1, left panel) and such requirements are met by pre-formed DHA available in breast milk (Figure 1, right panel). In this case, inter-individual variation in IQ due to genetically determined differences in DHA endogenous synthesis from metabolic precursors would only be observable in individuals who were not breastfed.^14^ Therefore, the effect of breastfeeding on intelligence would be stronger among carriers of genotypes associated with lower DHA endogenous synthesis compared with carriers of genotypes associated with higher synthesis, because the first depend more on breastfeeding to achieve optimal DHA levels for cognitive development. Importantly, our nutritional adequacy hypothesis postulates a weaker, but non-zero effect of breastfeeding on intelligence among carriers of DHA-increasing genotypes. because breastfeeding may act through many mechanisms in addition to providing pre-formed DHA.^1^^,^^17^

This G × E interaction has been investigated using single nucleotide polymorphisms (SNPs) in the *FADS2* gene.^14^^,^^18–21^ This gene encodes a desaturase enzyme that catalyzes a rate-limiting reaction in the LC-PUFAs pathway.^22^^,^^23^ Candidate gene and genome-wide approaches reported that minor alleles of SNPs in the *FADS2* gene were associated with lower levels of PUFAs in plasma and erythrocyte phospholipids.^24–27^ Caspi *et al*. were the first to evaluate the interaction between genetic variation in *FADS2* and breastfeeding, with IQ in children as the outcome.^18^ Two SNPs were evaluated: rs1535 (major/minor alleles: A/G) and rs174575 (major/minor alleles: C/G). These SNPs are in partial linkage disequilibrium, with an r^2^ metric ranging from 0.33 to 0.68 in European populations in 1000 Genomes Phase 3. For both SNPs, having ever being breastfed was positively associated with IQ in all genetic groups, except in G-allele homozygotes where there was no association.^18^ However, under the nutritional adequacy hypothesis outlined above, G-allele homozygotes are the subgroup expected to benefit the most from breastfeeding. This result is not only inconsistent with our hypothesis, but also with the notion that the benefits of breastfeeding on IQ exist (perhaps with varying magnitudes) in most population subgroups (in this case, genetically defined subgroups).

However, in a replication study, Steer *et al.* presented results consistent with our nutritional adequacy hypothesis, with breastfed individuals presenting similar mean values of IQ across *FADS2* genotypes.^14^ Such values were higher than those observed in never breastfed individuals, with the lowest value (and thus the greatest effect of breastfeeding) being in GG individuals.^14^ Morales *et al*.^28^ reported that a negative association between genotypes in other genetic variants related to lower activity of enzymes involved in elongation and desaturation processes and cognition, was only evident in non-breastfed individuals. Three studies in twins (but not twin studies, in the sense that they did not aim to estimate heritability) did not detect strong evidence supporting this G × E interaction.^19–21^

The contradictory results observed in the literature may be due to lack of statistical power and/or contextual differences that lead to heterogeneity between studies, as discussed in detail elsewhere.^29^ In this study, we aimed at improving the current understanding on this G × E interaction and gaining insights into the sources of heterogeneity between studies through a consortium-based initiative.^29^ Specifically, we addressed three research questions: (i) is breastfeeding positively associated with IQ in both subgroups of *FADS2* genotypes (i.e. G-allele homozygotes and others)?; (ii) is the association of breastfeeding with IQ different between subgroups of *FADS2* genotypes (i.e. is there a G × E interaction)?; and (iii) do study-level characteristics explain between-study heterogeneity in this G × E interaction?

## Methods

### Overview of the study protocol

The protocol of this study has been published elsewhere.^29^ Briefly, studies that were known by the coordinating team to have at least some of the data required, as well as other studies suggested by collaborators, were invited to participate. All studies that were contacted (and were eligible) accepted participation.

Eligibility criteria were: (i) availability of at least a binary breastfeeding variable (i.e. whether or not the study individuals were ever breastfed), intelligence measured using standard tests and at least rs174575 or rs1535 SNPs (either genotyped or imputed); and (ii) European ancestry studies, or multi-ethnic studies capable of defining a subsample of European ancestry individuals. Exclusion criteria were: (i) only poorly imputed genetic data available (metrics of imputation such as *r*^2^ or INFO quality below 0.3); (ii) twin studies; and (iii) lack of appropriate ethical approval.

Statistical analysis was performed locally by data analysts of the collaborating studies. Standardized analysis scripts written in R [http://www.r-project.org/] were prepared centrally and distributed to the analysts, along with a detailed analysis plan. The scripts automatically generated files containing summary descriptive and association statistics, which were centrally meta-analysed. As the study progressed, some modifications in the original protocol were required. These are described in detail in Supplementary Methods, available as Supplementary data at *IJE* online.

### Participating studies

A total of 10 eligible studies were identified, all of which were included in the meta-analysis: the 1982 Pelotas Birth Cohort Study,^30^^,^^31^ Dunedin Multidisciplinary Health and Development Study,^18^ Avon Longitudinal Study of Parents and Children (ALSPAC),^32^ Copenhagen Prospective Study on Asthma in Childhood (COPSAC) 2010,^33^^,^^34^ Generation R Study,^35–37^*INfancia y Medio Ambiente* (INMA) Project,^38^ Western Australian Pregnancy Cohort (Raine) Study,^39–41^*Småbørn Kost Og Trivsel*-I (SKOT-I),^42^^,^^43^ SKOT-II^44^^,^^45^ and Saguenay Youth Study (SYS).^46^^,^^47^

In an attempt to improve statistical power, a subsample of 32 842 individuals from the UK Biobank^48^ was included. These individuals had data on ever being breastfeed, intelligence measures and genetic data. However, this subsample did not fulfil the pre-established eligibility criteria because IQ was not measured using a standard test. Therefore, these data were used in secondary analyses only, and analyses including these data are clearly indicated as such. Information about the participating studies is shown in Supplementary Tables 1–3, available as Supplementary data at *IJE* online.

### Statistical analyses

The main outcome variable was IQ. IQ tests varied between studies (Supplementary Table 1, available as Supplementary data at *IJE* online). IQ measures were converted to Z scores (mean = 0 and variance = 1) within each participating study. The primary analysis involved breastfeeding (coded as never = 0 and ever = 1), *FADS2* polymorphism assuming a recessive genetic effect of the G allele (i.e. GG individuals = 1; heterozygotes and non-G allele homozygotes = 0) and an interaction term between them. Different genetic effects, different categorizations of breastfeeding, and exclusive breastfeeding (defined as receiving only breast milk and no other food or drink, including water) were evaluated in pre-planned secondary analyses. Unless explicitly stated, all analyses refer to any quality of breastfeeding (i.e. combining exclusive and non-exclusive breastfeeding).

Three analysis models were performed: (i) unadjusted (i.e. no covariates); (ii) adjusted 1: controlling for sex and age (linear and quadratic terms) when IQ was measured, ancestry-informative principal components (when available) and genotyping centre (for studies involving multiple laboratories); (iii) adjusted 2: same covariates in adjusted 1 model, as well as maternal education (linear and quadratic terms) and maternal cognition (linear and quadratic terms); if only one of the maternal variables was available, adjusted model 2 controlled only for that variable. Continuous covariates, as well as sex (which was coded as male = 0 and female = 1), were mean-centred before analysis, and squaring was performed before mean-centring. Covariate adjustment was performed by including not only a ‘main effect’ term, but also *FADS2* × covariate and breastfeeding × covariate interaction terms.^49^

As a sensitivity analysis, the role of gene-environment correlation was evaluated by repeating models (i) and (ii), but having maternal cognition (in Z scores) or maternal schooling (in years) as outcome variables rather than the participant’s IQ. Maternal cognition or schooling are important predictors of an individual’s IQ, and cannot be consequences of the participant’s genotype. Therefore, any evidence of breastfeeding × *FADS2* interaction in this analysis is indicative that those maternal variables may confound the main breastfeeding × *FADS2* interaction analysis having participant’s IQ as the outcome variable. This is a form of negative control analysis.^50^

Analyses were performed using linear regression with heteroskedasticity-robust standard errors. Results from all studies were pooled using fixed and random effects meta-analysis. Stratified meta-analysis and random effects meta-regression were used to evaluate the potential moderating role of the following variables (one meta-regression model per moderator): IQ test; adjustment for ancestry-informative principal components; age at IQ measurement; timing of breastfeeding measurement; continental region; mean year of birth; prevalence of having ever being breastfed; mean breastfeeding duration; and sample size. Adjusted R² values, which can be interpreted as the amount of between-study heterogeneity explained by the moderator, were obtained from the meta-regression models.

The power of our primary analysis, focusing on the rs174575 polymorphism, was quantified via simulations (see the Supplementary Material, available as Supplementary data at *IJE* online, for details). This complements the sample size calculations presented in the protocol by allowing inclusion of estimated between-study heterogeneity in the calculations.

## Results

### Characteristics of participating studies

As shown in Supplementary Table 1, available as Supplementary data at *IJE* online, seven out of the 10 eligible studies were conducted in Europe, four were population-based and two were multi-ethnic. The average year of birth ranged from 1972 to 2011. Three studies measured breastfeeding prospectively, and four studies (two in children and two in adults) measured IQ using the Wechsler Intelligence Scale.

Supplementary Table 2, available as Supplementary data at *IJE* online, provides a description of the two *FADS2* SNPs in each study. The SNPs rs174575 and rs1535 were directly genotyped in three and five studies, respectively. The minimum value of imputation quality was 0.984. The frequency of the G allele ranged from 20.5% to 30.8% for the rs174575 variant, and from 28.5% to 39.1% for the rs1535 variant. There was no strong statistical evidence against Hardy-Weinberg equilibrium, with the smallest *P*-values being 0.058 (Generation R) and 0.074 (SKOTI-II) for rs174575, and 0.085 (1982 Pelotas Birth Cohort), 0.044 (Raine) and 0.089 (SKOTI-II) for rs1535. Although these results may be suggestive of some population substructure (especially in Generation R and in the 1982 Pelotas Birth Cohort, which are multi-ethnic studies) or batch effects (especially in SKOTI-II, which is a combination of two independent studies), it is unlikely that such phenomena substantially influenced the results because ancestry-informative principal components, computed using genome-wide genotyping data, were adjusted for in these four studies.

Additional study characteristics are displayed in Supplementary Table 3, available as Supplementary data at *IJE* online. Among eligible studies (i.e. excluding the UK Biobank), the mean age, maternal education and breastfeeding duration ranged from 2.5 to 30.2 years, 11 to 19 years and 2.3 to 8.2 months, respectively. Each sex represented approximately half of the participants in all studies. All IQ measures produced a variable with mean close to 100 and similar standard deviations (median: 12.2; range: 9.6 to 16.3). The exception was the one used in SKOT-I and SKOT-II (i.e. third edition of the Ages and Stages Questionnaire), which produced a variable with mean close to 50. The median of the prevalence of ever breastfeeding was 91% (ranging from 50% to 95%), and the median of the median of any breastfeeding duration was 4.3 months (ranging from 0 to 7.9). Among all individuals included in primary analysis for at least one of the SNPs (*n* = 13 292), 11 055 were ever breastfed.

### Primary analysis

In analyses without stratification according to *FADS2* genotype, ever breastfeeding was associated with increases of 0.37 (95% CI: 0.32; 0.42) and 0.30 (95% CI: 0.20; 0.40) Z scores in IQ in fixed and random effects meta-analyses, respectively. Assuming that IQ has a standard deviation (SD) of 12.2 points (the median of the standard deviation of IQ measures among participating studies), these coefficients correspond to 4.5 and 3.7 points in IQ, respectively. In the fully adjusted model (adjusted 2), the respective coefficients were 0.26 (95% CI: 0.21; 0.32) and 0.17 (95% CI: 0.03; 0.32), or 3.2 and 2.1 points in IQ.

**Figure 2.**
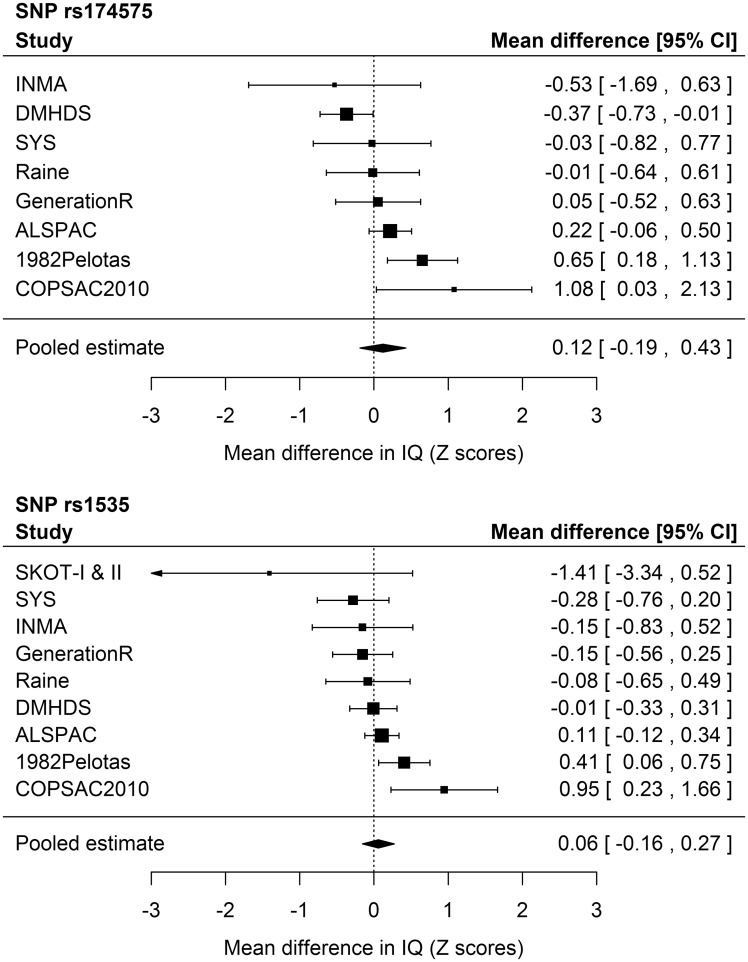
Forest plots of the G × E interaction coefficients^a^ from the fully-adjusted^b^ primary analysis (*FADS2* variants coded in recessive form, and breastfeeding categorized into ever x never breastfeeding) based on random effects meta-analysis. SKOT-I and SKOT-II were excluded from the analyses for the rs174575 polymorphism because the model did not fit (due to a combination of modest sample size, high prevalence of breastfeeding and assuming a recessive genetic effect of the rarest allele). 1982Pelotas, 1982 Pelotas Birth Cohort; ALSPAC, Avon Longitudinal Study of Parents and Children; COPSAC2010, Copenhagen Prospective Study on Asthma in Childhood 2010; DMHDS, Dunedin Multidisciplinary Health and Development Study; GenerationR, Generation R Study; INMA, INfancia y Medio Ambiente [Environment and Childhood]; Raine, Western Australian Pregnancy Cohort (Raine) Study; SKOT-I and II, Småbørn Kost Og Trivsel (I and II); SYS, Saguenay Youth Study. ^a^These coefficients can be interpreted as the mean difference in IQ Z scores comparing ever with never breastfed individuals among GG carriers minus the mean difference in IQ Z scores comparing ever with never breastfed individuals among carriers of other genotypes. ^b^Covariates were sex, age (linear and quadratic terms), ancestry-informative principal components (if available), genotyping centre (if necessary), maternal education (linear and quadratic terms) and/or maternal cognition (linear and quadratic terms).

Table 1 and Figure 2 display the results of the primary analysis. There was considerable between-study heterogeneity. Among non-G carries for the rs174575 SNP, pooled random effects estimates of mean differences in IQ Z scores according to breastfeeding (ever = 1; never = 0) were 0.29 (95% CI: 0.17; 0.40) and 0.15 (95% CI: 0.00; 0.31) in the unadjusted and fully-adjusted models, respectively. Among GG individuals, the respective estimates were 0.43 (95% CI: 0.16; 0.70) and 0.31 (95% CI: 0.05; 0.58). There was no strong evidence of interaction, with pooled estimates of the breastfeeding × *FADS2* interaction term of 0.18 (95% CI: −0.18; 0.54) and 0.12 (95% CI: −0.19; 0.43), respectively. These coefficients can be interpreted as the mean difference in IQ Z scores comparing ever with never breastfed individuals among GG carriers, minus the mean difference in IQ Z scores comparing ever with never breastfed individuals among carriers of other genotypes—e.g. a positive interaction coefficient indicates that the benefit of breastfeeding on IQ is stronger among GG individuals. Similar results were obtained when using fixed effects meta-analysis.

**Table 1.** Meta-analytical linear regression coefficients (β) of cognitive measures (in standard deviation units) according to breastfeeding (never = 0; ever = 1), within strata of *FADS2* rs174575 or rs1513 genotypes (recessive effect)

SNP	Coefficient	Model	Number of		Fixed effects meta-analysis			Random effects meta-analysis			
			Estimates	Subjects	β	95% CI	*P*-value	β	95% CI	*P*-value	I^2^ (%)
rs174575	β in C-allele	Unadjusted	8	11 741	0.37	0.32; 0.41	8.6 × 10^−50^	0.29	0.17; 0.40	7.6 × 10^−7^	76.4
	carriers	Adjusted (1)^a^	8	11 719	0.37	0.32; 0.42	7.7 × 10^−48^	0.29	0.18; 0.41	7.9 × 10^−7^	74.2
		Adjusted (2)^b^	8	11 241	0.25	0.20; 0.31	6.4 × 10^−20^	0.15	0.00; 0.31	0.055	84.1
	β in GG	Unadjusted	8	873	0.43	0.28; 0.58	3.8 × 10^−8^	0.43	0.16; 0.70	0.002	64.4
	individuals	Adjusted (1)^a^	8	871	0.39	0.23; 0.54	9.3 × 10^−7^	0.35	0.04; 0.65	0.024	67.2
		Adjusted (2)^b^	8	836	0.34	0.17; 0.51	6.4 × 10^−5^	0.31	0.05; 0.58	0.020	47.4
	G×E	Unadjusted	8	12 614	0.11	−0.05; 0.27	0.188	0.18	−0.18; 0.54	0.323	77.6
		Adjusted (1)^a^	8	12 590	0.04	−0.12; 0.21	0.603	0.07	−0.29; 0.43	0.705	75.5
		Adjusted (2)^b^	8	12 077	0.10	−0.07; 0.28	0.244	0.12	−0.19; 0.43	0.445	59.5
rs1535	β in A-allele	Unadjusted	9	11 690	0.37	0.32; 0.42	9.2 × 10^−49^	0.29	0.18; 0.40	4.6 × 10^−7^	73.5
	carriers	Adjusted (1)^a^	9	11 666	0.37	0.32; 0.42	9.9 × 10^−47^	0.29	0.16; 0.42	7.1 × 10^−6^	76.0
		Adjusted (2)^b^	9	11 186	0.26	0.20; 0.31	1.9 × 10^−19^	0.15	−0.01; 0.32	0.065	84.0
	β in GG	Unadjusted	9	1512	0.29	0.17; 0.41	2.2 × 10^−6^	0.24	0.05; 0.43	0.013	54.1
	individuals	Adjusted (1)^a^	9	1509	0.33	0.20; 0.45	2.2 × 10^−7^	0.27	0.08; 0.47	5.4 × 10^−3^	47.7
		Adjusted (2)^b^	9	1447	0.28	0.16; 0.41	1.2 × 10^−5^	0.25	0.09; 0.41	0.003	25.9
	G×E	Unadjusted	9	13 202	−0.03	−0.16; 0.10	0.663	−0.04	−0.24; 0.15	0.646	42.6
		Adjusted (1)^a^	9	13 175	−0.02	−0.16; 0.11	0.720	−0.03	−0.28; 0.21	0.778	60.9
		Adjusted (2)^b^	9	12 633	0.07	−0.06; 0.21	0.277	0.06	−0.16; 0.27	0.592	49.6

GxE, interaction between breastfeeding and polymorphisms in the *FADS2* gene; *n*_estimates_, number of estimates being pooled; *n*_subjects_, pooled sample size.

a Covariates were sex, age (linear and quadratic terms), ancestry-informative principal components (if available) and genotyping centre (if necessary).

b Same covariates as in the adjusted (1) model, in addition to maternal education (linear and quadratic terms) and/or maternal cognition (linear and quadratic terms).

Results for the rs1535 variant presented a similar trend, but were even less suggestive of interaction. When using random effects meta-analysis, the estimates of the interaction term were −0.04 (95% CI: −0.24; 0.15) in the unadjusted and 0.06 (95% CI: −0.16; 0.27) in the fully-adjusted model (i.e. adjusted model 2). Using fixed effects meta-analysis yielded similar results.

### Secondary analysis

As shown in Table 2 and Supplementary Tables 4–6, available as Supplementary data at *IJE* online, there was no strong indication of interaction when analysing other categorizations of breastfeeding duration and *FADS2* SNPs coded assuming a recessive effect. This was also the case when *FADS2* variants were coded assuming additive (Supplementary Table 7, available as Supplementary data at *IJE* online), dominant (Supplementary Table 8, available as Supplementary data at *IJE* online) and overdominant (Supplementary Table 9, available as Supplementary data at *IJE* online) effects. The same was observed for exclusive breastfeeding (Supplementary Tables 10–13, available as Supplementary data at *IJE* online).

**Table 2.** Meta-analytical linear regression coefficients (β) of the interaction term between *FADS2* rs174575 or rs1535 genotypes (recessive effect) with breastfeeding (<6 months vs ≥6 months, in ordinal categories or in months), having cognitive measures (in standard deviation units) as the outcome

SNP	Breastfeeding	Model	Number of		Fixed effects meta-analysis			Random effects meta-analysis			
	categorization		Estimates	Subjects	β	95% CI	*P*-value	β	95% CI	*P*-value	I^2^ (%)
rs174575	<6 months = 0	Unadjusted	8	11 733	0.05	−0.10; 0.20	0.515	0.04	−0.14; 0.22	0.647	23.1
	≥6 months = 1	Adjusted (1)^a^	8	11 706	0.07	−0.09; 0.23	0.378	0.08	−0.18; 0.35	0.546	53.6
		Adjusted (2)^b^	8	11 242	0.10	−0.07; 0.26	0.244	0.17	−0.32; 0.65	0.496	82.6
	Numerically-	Unadjusted	8	11 733	0.04	−0.01; 0.09	0.104	0.06	−0.02; 0.15	0.150	57.1
	coded duration	Adjusted (1)^a^	8	11 706	0.04	−0.02; 0.09	0.189	0.06	−0.05; 0.16	0.282	58.7
	categories	Adjusted (2)^b^	8	11 242	0.04	−0.01; 0.10	0.132	0.09	−0.09; 0.26	0.346	84.6
	Duration, in	Unadjusted	8	11 733	0.01	−0.01; 0.02	0.371	0.01	−0.01; 0.03	0.335	13.9
	months	Adjusted (1)^a^	8	11 706	0.00	−0.01; 0.02	0.608	0.01	−0.02; 0.04	0.635	63.3
		Adjusted (2)^b^	8	11 242	0.00	−0.01; 0.02	0.782	0.01	−0.04; 0.07	0.602	85.3
rs1535	<6 months = 0	Unadjusted	8	12 018	−0.05	−0.17; 0.08	0.460	−0.05	−0.17; 0.08	0.460	0.0
	≥6 months = 1	Adjusted (1)^a^	8	11 991	−0.07	−0.20; 0.05	0.248	−0.07	−0.20; 0.06	0.302	8.0
		Adjusted (2)^b^	8	11 499	−0.08	−0.21; 0.04	0.194	−0.08	−0.21; 0.05	0.216	3.9
	Numerically-	Unadjusted	8	12 018	0.00	−0.04; 0.04	0.966	0.00	−0.04; 0.04	0.966	0.0
	coded duration	Adjusted (1)^a^	8	11 991	−0.01	−0.06; 0.03	0.508	−0.02	−0.09; 0.05	0.635	54.3
	categories	Adjusted (2)^b^	8	11 499	−0.01	−0.05; 0.03	0.675	−0.01	−0.07; 0.05	0.728	29.9
	Duration, in	Unadjusted	8	12 018	0.00	−0.01; 0.01	0.805	0.00	−0.01; 0.01	0.805	0.0
	months	Adjusted (1)^a^	8	11 991	0.00	−0.01; 0.01	0.538	−0.01	−0.03; 0.01	0.330	59.6
		Adjusted (2)^b^	8	11 499	−0.01	−0.02; 0.01	0.320	−0.01	−0.02; 0.01	0.344	35.5

*n*_estimates_, number of estimates being pooled; *n*_subjects_, pooled sample size.

a Covariates were sex, age (linear and quadratic terms), ancestry-informative principal components (if available) and genotyping centre (if necessary).

b Same covariates than in the adjusted (1) model, in addition to maternal education (linear and quadratic terms) and/or maternal cognition (linear and quadratic terms).

Supplementary Table 14, available as Supplementary data at *IJE* online, displays the results obtained when including the UK Biobank, which was analysed as two independent samples according to the genotyping platform (Biobank_Axiom and Biobank_BiLEVE). Its inclusion resulted in a combined sample size of more than 45 000 individuals. When *FADS2* variants were coded assuming recessive effects, the pooled estimates from the unadjusted model were −0.02 (95% CI: −0.10; 0.06) and 0.08 (95% CI: −0.13; 0.29) for fixed and random effects meta-analysis, respectively. The corresponding estimates from the adjusted (1) model were −0.04 (95% CI: −0.13; 0.04) and 0.00 (95% CI: −0.21; 0.20), respectively. There was also no strong statistical evidence supporting an interaction when other genetic effects were assumed.

### Sensitivity analysis and power calculation

Table 3 displays the results of random effects meta-regression. Neither type of IQ test, timing of breastfeeding measurement, continental region nor mean year of birth explained a substantial amount of between-study heterogeneity. For the rs174575 variant, the adjusted R² of ancestry-informative principal components was 88.0%, with pooled estimates of 0.28 (95% CI: 0.02; 0.54) and −0.38 (95% CI: −0.72; −0.04) Z scores in IQ from studies that did and did not adjust for principal components, respectively, which would be suggestive of confounding due to population stratification towards a negative association. Age at IQ measurement was inversely associated with the magnitude of the interaction term, with pooled estimates of 0.06 (95% CI: −0.46; 0.58) and 0.20 (95% CI: −0.18; 0.58) when IQ was measured at 10 years of age or more, or before that age, respectively, possibly suggesting an attenuation of the effect over time. The adjusted R² was 10.4% when entering age as a continuous variable, but 0% when dichotomized. When stratifying studies according to prevalence of ever breastfeeding, the pooled estimate among studies with a prevalence ≥90% was 0.36 (95% CI: −0.19; 0.90), and −0.04 (95% CI: −0.38; 0.29) when pooling the remaining studies. Adjusted R² estimates were 16.4% and 72.3% when prevalence of ever breastfeeding was analysed as a binary and as a continuous variable, respectively. Among studies with breastfeeding duration equal to or greater than the median among studies (i.e. 5.85 months), the pooled estimate was 0.50 (95% CI: −0.06; 1.06), compared with 0.14 (95% CI: −0.10; 0.38) when pooling the remaining studies. The adjusted R² was 45.5% when breastfeeding duration was dichotomized at the median, but 0% when analysed continuously. When stratifying studies into larger (≥1000 individuals) and smaller (<1000 individuals), the pooled estimates were 0.26 (95% CI: 0.00; 0.52) and −0.03 (95% CI: −0.63; 0.56), with an adjusted R² of 33.8% when sample size was dichotomized, and of 0% when analysed in continuous form.

**Table 3.** Stratified random effects meta-analytical linear regression coefficients (β) of the interaction term between *FADS2* rs174575 or rs1535 genotypes (recessive effect) with breastfeeding (never = 0; ever = 1), having cognitive measures (in standard deviation units) as the outcome. Estimates from the fully adjusted model were used

Analysis	Categories	rs174575 (CC or CG = 0; GG = 1)				rs1535 (AA or AG = 0; GG = 1)			
stratified by		*n*_estimates_	β (95% CI)	*P*-value	Adjusted	*n*_estimates_	β (95% CI)	*P-*value	Adjusted
		(*n*_subjects_)			R² (%)	(*n*_subjects_)			R² (%)
IQ test	Wechsler^a^	8055 (4)	0.12 (−0.32; 0.56)	0.591	0.0	8070 (4)	0.09 (−0.14; 0.32)	0.452	0.0
	Other	4022 (4)	0.12 (−0.37; 0.61)	0.631		4563 (5)	0.02 (−0.45; 0.49)	0.932	
Adjustment	Yes	10 441 (6)	0.28 (0.02; 0.54)	0.036	88.0	10753 (7)	0.09 (−0.19; 0.37)	0.531	0.0
for PCs	No	1636 (2)	−0.38 (−0.72; −0.04)	0.028		1880 (2)	−0.03 (−0.32; 0.25)	0.814	
Age at IQ	≥10 years	4373 (4)	0.06 (−0.46; 0.58)	0.825	0.0^b^; 10.4^c^	4374 (4)	0.04 (−0.25; 0.34)	0.773	0.0^b^; 0.0^c^
measurement	<10 years	7704 (4)	0.20 (−0.18; 0.58)	0.304		8259 (5)	0.07 (−0.31; 0.45)	0.700	
BF measurement	Prospective	6912 (3)	0.27 (−0.10; 0.63)	0.155	0.0	6926 (3)	0.20 (−0.25; 0.64)	0.383	0.0
	Retrospective	5165 (5)	−0.01 (−0.48; 0.47)	0.979		5707 (6)	−0.01 (−0.28; 0.27)	0.951	
Continental	Europe	7704 (4)	0.20 (−0.18; 0.58)	0.304	0.0	8259 (5)	0.07 (−0.31; 0.45)	0.700	0.0
region	Other	4373 (4)	0.06 (−0.46; 0.58)	0.825		4374 (4)	0.04 (−0.25; 0.34)	0.773	
Mean year of	≥2000	3002 (3)	0.20 (−0.58; 0.98)	0.616	0.0^b^; 2.9^c^	3543 (4)	0.03 (−0.62; 0.69)	0.917	0.0^b^; 0.0^c^
birth	<2000	9075 (5)	0.10 (−0.27; 0.46)	0.601		9090 (5)	0.07 (−0.13; 0.27)	0.469	
Prevalence of	≥90	4798 (4)	0.36 (−0.19; 0.90)	0.200	16.4^b^; 72.3^c^	5339 (5)	0.15 (−0.31; 0.62)	0.519	0.0^b^; 8.3^c^
any BF (%)	<90	7279 (4)	−0.04 (−0.38; 0.29)	0.803		7294 (4)	0.01 (−0.15; 0.18)	0.869	
Duration of any	≥5.85	3367 (3)	0.50 (−0.06; 1.06)	0.081	45.5^b^; 0.0^c^	3665 (4)	0.27 (−0.28; 0.82)	0.333	22.2^b^; 4.9^c^
BF (months)	<5.85	7866 (4)	0.14 (−0.10; 0.38)	0.255		8123 (4)	−0.01 (−0.19; 0.16)	0.882	
Sample size (*n*)	≥1000	9177 (4)	0.26 (0.00; 0.52)	0.052	33.8^b^; 0.0^c^	9191 (4)	0.11 (−0.12; 0.34)	0.365	0.0^b^; 0.0^c^
	<1000	2900 (4)	−0.03 (−0.63; 0.56)	0.910		3442 (5)	0.01 (−0.43; 0.45)	0.974	

PCs, ancestry-informative genetic principal components; BF, breastfeeding; *n*_estimates_, number of estimates being pooled; *n*_subjects:_ pooled sample size.

a Includes both Wechsler Adult Intelligence Scale (ALSPAC and Dunedin Multidisciplinary Health and Development Study) and Wechsler Intelligence Scale for Children (1982 Pelotas Birth Cohort and Saguenay Youth Study).

b Variable categorized as shown in the table.

c Variable entered in continuous form (e.g. age at outcome measurement modelled in years, as a continuous variable).

Regarding the rs1535 variant, the following subgroup-specific estimates were consistent with those of the rs174575 SNP: adjustment for principal components, with pooled estimates of 0.09 (95% CI: −0.19; 0.37) and −0.03 (95% CI: −0.32; 0.25) among studies that did and did not perform this adjustment, respectively; age at IQ measurement, with pooled estimates of 0.04 (95% CI: −0.19; 0.37) and 0.07 (95% CI: −0.31; 0.45) among studies that measured IQ when individuals were ≥10 and <10 years old, respectively; and sample size, with pooled estimates of 0.11 (95% CI: −0.12; 0.34) and 0.01 (95% CI: −0.43 and 0.45) among larger and smaller studies, respectively. However, in all those cases the adjusted R² values were 0%. Prevalence of ever breastfeeding presented adjusted R² values of 0% and 8.3% when dichotomized and when analysed continuously, respectively. The pooled estimates for the rs1535 variant were 0.15 (95% CI: −0.31; 0.62) and 0.01 (95% CI: −0.15; 0.18) among studies with prevalences of ever breastfeeding of ≥90% and <90%, respectively. The most consistent moderator between SNPs was breastfeeding duration, with pooled estimates for the rs1535 SNP of 0.27 (95% CI: −0.28; 0.82) and −0.01 (95% CI: −0.19; 0.16) among studies with ≥5.85 and <5.85 months of average duration, respectively; adjusted R² values were 22.2% and 4.9% when breastfeeding duration was dichotomized and analysed continuously, respectively.

There was no strong evidence in support of gene-environment correlation involving maternal education or maternal cognition (Table 4). Regarding the rs174575 variant, random effects meta-analytical estimates from the adjusted model were 0.16 (95% CI: −0.45; 0.78) for maternal education, and −0.02 (95% CI: −0.25; 0.21) for maternal cognition, respectively. The corresponding estimates for the rs1535 SNP were −0.12 (95% CI: −0.51; 0.27) and 0.14 (95% CI: −0.04; 0.33).

**Table 4.** Meta-analytical linear regression coefficients (β) of the interaction term between *FADS2* rs174575 or rs1535 genotypes (recessive effect) with breastfeeding (never = 0; ever = 1), having maternal education (in complete years) or maternal cognitive measures (in standard deviation units) as the outcome

SNP	Outcome	Model	Number of		Fixed effects meta-analysis			Random effects meta-analysis			
			Estimates	Subjects	β	95% CI	*P*-value	β	95% CI	*P*-value	I^2^ (%)
rs174575	Maternal	Unadjusted	7	14 671	0.28	−0.11; 0.66	0.159	0.59	−0.72; 1.91	0.375	81.1
	education	Adjusted (1)^a^	7	12 113	0.16	−0.31; 0.62	0.509	0.16	−0.45; 0.78	0.607	14.1
	Maternal	Unadjusted	5	6299	0.10	−0.10; 0.31	0.326	0.10	−0.13; 0.33	0.389	18.1
	cognition	Adjusted (1)^a^	5	6126	−0.02	−0.25; 0.21	0.854	−0.02	−0.25; 0.21	0.854	0.0
rs1535	Maternal	Unadjusted	8	15 447	−0.05	−0.38; 0.28	0.784	−0.04	−0.39; 0.31	0.814	1.4
	education	Adjusted (1)^a^	8	12 743	−0.12	−0.51; 0.27	0.540	−0.12	−0.51; 0.27	0.540	0.0
	Maternal	Unadjusted	5	6556	0.10	−0.08; 0.28	0.272	0.10	−0.08; 0.28	0.272	0.0
	cognition	Adjusted (1)^a^	5	6378	0.14	−0.05; 0.33	0.160	0.14	−0.05; 0.33	0.160	0.0

*n*_estimates_, number of estimates being pooled; *n*_subjects_ , pooled sample size.

a Covariates were sex, age (linear and quadratic terms), ancestry-informative principal components (if available) and genotyping centre (if necessary).

Supplementary Table 15, available as Supplementary data at *IJE* online, displays the results of the power calculations. Assuming no between-study heterogeneity, the power of the primary analysis was ≥80% to detect a G × E coefficient between 0.219 and 0.263 IQ Z scores (with the latter corresponding to the point estimate reported by Steer *et al*.,^14^ which is the largest previous study on this topic) using fixed effects meta-analysis. The random effects model was similarly powered to detect G × E coefficients between 0.263 and 0.307. Assuming a between-study variance of 0.103 (which was the observed between-study heterogeneity in our meta-analysis), the fixed effects model had ≥80% of power to detect G × E coefficients between 0.307 and 0.351, whereas the random effects model was similarly powered for coefficients between 0.439 and 0.483. Of note, in all cases power to detect our point G × E estimate of 0.121 was <50%.

## Discussion

In this study, we assessed the hypothesis that *FADS2* polymorphisms modify the association between breastfeeding and IQ, as predicted by a nutritional adequacy hypothesis (Figure 1). Our primary analyses were not supportive of this interaction. This was also the case in a priori secondary analyses using different categorizations of breastfeeding, exclusive rather than any quality of breastfeeding, assuming different genetic effects and including a large study that did not meet all eligibility criteria. Sensitivity analyses were not supportive that gene-environment correlation involving maternal education or maternal cognition substantially influenced the results. Random effects meta-regression suggested that breastfeeding duration was an important moderator.

Results from our primary and secondary analyses were not supportive of the nutritional adequacy hypothesis, according to which a positive interaction coefficient would be expected.^14^ In other words, there might be no upper limit (or it may be very high) of the effects of LC-PUFAs on IQ, so that supplementing infants with LC-PUFAs could be beneficial for cognition for both lactating and non-lactating infants alike. Importantly, this does not imply that LC-PUFAs supplementation completely replaces the benefits of breastfeeding, since the latter may act through diverse mechanisms, and also provide benefits other than for intelligence.^1^^,^^17^ Importantly, the absence of strong evidence of G × E interaction corroborates the notion that there are cognitive benefits of breastfeeding in both genetic subgroups of *FADS2* genotypes, which were also seen in the primary analysis (which were stratified on genotype). This finding is against the notion that there is a *FADS2* genetic subgroup where breastfeeding is not associated with IQ (which was one of the findings of the first study on this G × E interaction^18^) and is in accordance with the notion that breastfeeding is beneficial to most population subgroups.

On the other hand, in our random effects meta-regression analysis, studies with longer average breastfeeding duration generally presented interaction coefficients that were positive and stronger in magnitude than studies with shorter breastfeeding duration. Moreover, average breastfeeding duration was the most consistent moderator between polymorphisms (Table 3). Considering that positive interaction coefficients are expected under the nutritional adequacy hypothesis, this result raises the possibility that there may be an upper limit of the benefits of LC-PUFAs, but achieving such limits from breast milk requires that breastfeeding lasts for some currently unknown time. Given that breastfeeding practices in the participating studies were generally well below international recommendations,^51^^,^^52^ it is possible that the amount of LC-PUFA received from breast milk were, on average, lower than this threshold. However, the moderating effect of average breastfeeding duration was not a statistically robust finding and could be due to chance, especially given the large number of moderators evaluated in the meta-regression analysis.

The strengths of our study include: appropriate sample size for the primary analysis^29^; publication of the study protocol,^29^ which helps to avoid biased reporting; analyses performed using standardized analysis scripts and datasets harmonized as much as possible; inclusion of published and unpublished reports, thus minimizing publication bias; several a priori defined secondary and sensitivity analyses; proper adjustment for covariates in the G × E setting; and IQ measures with similar variances, which reduces heterogeneity that could arise due to Z score conversion.^53^^,^^54^

Our study also had limitations. Some of them relate to the small numbers of individuals in some categories, which we addressed by adapting the protocol, such as by re-defining the ‘never breastfed’ group and by excluding some categories of breastfeeding from the analysis (see Supplementary Methods, available as Supplementary data at *IJE* online for detailed descriptions of all adaptations in the protocol). Other limitations were: small sample size for some analyses, such as those involving exclusive breastfeeding; heterogeneity in important study characteristics, such as age (including pooling children and adult studies), type of IQ test and timing of breastfeeding measurement; and small number of studies in the meta-regression analyses. Another potential limitation was lack of adjustment for maternal genotypes, which may confound the association between participant’s genotype and IQ because maternal genotypes may influence fatty acid composition in breast milk.^28^ However, although there is evidence that this may be the case for some genetic variants implicated in LC-PUFA metabolism,^28^ previous studies found no strong evidence that maternal genotypes (rs174575 and rs1535) were associated with offspring’s IQ or that maternal genotypes interact with breastfeeding.^14^^,^^18^ It is also possible that there are epistatic (i.e. gene-gene interaction) relationships between genes implicated in this pathway. This could mean that focusing only on two variants in a single gene may not capture the whole complexity of the interplay between breastfeeding and genetic influences of LC-PUFA levels on cognitive development.

Another potential limitation is the fact that non-breastfed individuals may have received formula fortified with LC-PUFAs. This could attenuate the G × E interaction coefficient even if the nutritional adequacy hypothesis is true, because LC-PUFA requirements are being achieved through formula. However, as shown in Table 4, the majority of the individuals included in the primary analysis were born before the year 2000, and widespread LC-PUFAs fortification began in the early 2000s.^55^ Moreover, mean birth year did not substantially explain between-study heterogeneity. Finally, simply adding nutrients present in breast milk to formula does not necessarily mimic the biological effects of such nutrients in breast milk,^56^ because the benefits of the latter depend on a complex balance between its various components.^1^

A final limitation of our study is lack of power to detect G × E coefficients of relevant magnitude (Supplementary Table 15, available as Supplementary data at *IJE* online). We believe there are three main reasons for this. One of them is substantial between-study heterogeneity, which was mitigated both by design (e.g. only individuals of European ancestry measured using standard tests were included) and analysis (harmonized data preparation and analysis), but could not be fully eliminated (as described above). There is a trade-off between heterogeneity and sample size, and in the case of this study, including further restrictions in the eligibility criteria would be prohibitive. The second is overestimation of the G × E coefficient in the sample size calculations, where we used the point estimate reported by Steer *et al*.,^14^ which is the largest previous study on this topic, as the most likely value. The point estimate from our primary analysis (fully adjusted model) involving the rs174575 variant was about half of Steer and colleagues’ result. Finally, our meta-regression results indicate that coding breastfeeding into a never vs ever variable is unlikely to be appropriate, which might also have contributed to power loss. Given these power issues, our most robust conclusion is that the ‘nullifying’ effect of the G-allele on the association between breastfeeding and IQ is unlikely to exist, given that breastfeeding was positively associated with IQ in both genetic groups (Table 1).

Although our primary findings were not supportive of an interaction between breastfeeding and *FADS2* polymorphisms, random effects meta-regression results suggest that a modified form of such interaction may exist, because studies with longer average breastfeeding duration generally presented stronger positive estimates. Given the aforementioned limitations of the meta-regression analysis, such interaction should be investigated in future studies comparing different categories of breastfeeding duration rather than simply never vs ever comparisons (or other categorizations used here). Since such analysis would involve many subgroupings, the best alternative would likely be to perform them in a large dataset of individual-level data, which may be achieved by a consortium-based effort such as this collaborative meta-analysis. This and other future investigations will be important to further refine our understanding on the role of LC-PUFAs on the association between breastfeeding and intelligence. This will also have practical implications, such as identifying whether current breastfeeding recommendations provide for the upper limit of cognitive benefits related to LC-PUFAs intake (if such limit exists), and the potential benefits (if any) of supplementing a lactating infant with LC-PUFAs.

## Funding

This work was supported by several funding agencies: see the Supplementary material (available as Supplementary data at *IJE* online) for study-specific funders and grant numbers. This work was coordinated by researchers working within the Medical Research Council (MRC) Integrative Epidemiology Unit, which is funded by the MRC and the University of Bristol (MC_UU_12013/1, MC_UU_12013/9, MC_UU_00011/1).

## Supplementary Material

Supplementary Material

Supplementary Tables
