# Supplementary material for: Effect modification of FADS2 polymorphisms on the association between breastfeeding and intelligence: results from a collaborative meta-analysis
Source: Int J Epidemiol. 2018 Dec 11;48(1):45–57. doi: 10.1093/ije/dyy273 (PMC6380307; doi:10.1093/ije/dyy273)
Supplement: Supplementary Material [file dyy273_supplementary_material.docx]

**Supplementary Material**

Summary

[Supplementary Methods 2](#_Toc516675727)

[Study Acknowledgements 5](#_Toc516675728)

[References 10](#_Toc516675729)

# Supplementary Methods

**Modifications in the study protocol**

After the publication of the protocol, some revisions in the analysis plan were necessary. They were performed after evaluating descriptive statistics, but before pooling study-level regression coefficients. In addition to the inclusion of a study (UK Biobank) that did not achieve all eligibility criteria (as explained in the main text), the revisions were:

i) Combination of SKOT-I and SKOT-II into a single study.

SKOT-I and SKOT-II were the studies with the smallest number of participants. Their main difference is that SKOT-II included only obese (pre-pregnancy BMI>30 kg/m²) mothers. Due to the small number of participants (likely accentuated by the very high prevalence of breastfeeding), in some analysis the model failed to converge, thus preventing these studies from contributing. To overcome this, both studies were combined into a single sample.

ii) Re-definition of never being breastfed.

In two studies (COPSAC 2010 and SKOT-I & II) the prevalence of never being breastfed was <1% (Supplementary Table 3). This was an issue especially because these studies were not large (551 and 299 individuals, respectively), the analyses involve fitting an interaction term, and the primary analysis assumes a recessive effect of the rarest allele. Therefore, in those studies, the binary variables of never vs. ever breastfeeding (for both any quality and exclusive) were re-defined as follows: 0: never breastfed or breastfed for less than 1 month; 1: breastfed for at least 1 month.

iii) Re-definition of exclusive breastfeeding.

Data on exclusive breastfeeding was unavailable in the 1982 Pelotas Birth Cohort, INMA, RAINE and SKOT-I & II studies. Those used predominant breastfeeding instead.

iv) Exclusion of the ordinal breastfeeding (for both any quality and exclusive) variable.

In the study protocol, one of the breastfeeding variables was an ordinal variable coded as follows: 0: none; 1: 0.01-1.00 months; 2: 1.01-3.00 months; 3: 3.01-6.00 months; 4: >6.00 months. After evaluating descriptive statistics, we noted that some categories (especially regarding exclusive breastfeeding) had very few individuals (Supplementary Table 3). Information on breastfeeding duration was not available for one eligible study (nor for the UK Biobank subsample), and in only two of the remaining studies the median of breastfeeding duration was at least six months. This was an issue due to the same reasons explained above, so we opted for removing this variable. However, this same variable coded numerically (i.e., assuming a linear trend) was maintained.

v) Exclusion of the exclusive breastfeeding dichotomised into <6 months vs. ≥6 months.

The pre-planned analyses described in the protocol included analyses of exclusive breastfeeding in four different categorisations: never vs. ever; ordinal variable of breastfeeding duration (coded assuming a linear effect); exclusive breastfeeding duration, in months; and <6 vs. ≥6 months. However, in studies with information on exclusive breastfeeding duration, fewer than 2% of all the children (Supplementary Table 3) were breastfed exclusively for more than 6 months. This was an issue due to the same reasons explained above, so we opted for removing this variable.

vi) Additional moderators in meta-regression analysis.

In the study protocol, it was specified that the following variables would be studied as moderators in meta-regression analyses: IQ test, adjustment for ancestry-informative principal components, age when IQ was measured, timing of breastfeeding measurement, continental region, prevalence of having ever being breastfed and mean breastfeeding duration. After publishing the protocol, we decided to also include average year of birth of study participants and sample size of each study.

vii) Not adjusting for maternal cognition in the ALSPAC study.

Apart from the UK Biobank, ALSPAC was the largest study included in this meta-analysis, with >4700 individuals in the unadjusted model of the primary analysis. Both maternal education and maternal cognition were available, but the latter was measured in less than 2000 of the individuals included in the primary analysis. To avoid such substantial sample size loss and given that education is highly correlated with cognitive measures, we opted for adjusting ALSPAC estimates only for maternal education in the “adjusted 2” model (in addition to the covariates adjusted for in the “adjusted 1” model). However, ALSPAC still contributed to the sensitivity analysis that had maternal cognition as the outcome variable.

**Power calculations**

We used simulations to calculate the power of our primary analysis to detect different values of the G×E (in this case, *FADS2*×Breastfeeding) coefficient. We focused on the rs174575 variant, for which G×E coefficients and standard errors were available for eight studies (excluding UK Biobank, which was not included in the primary analysis), as shown in Table 1 and Figure 2. The model used to generate summary data was:

$$\hat{\beta}_{j}=N\left( \beta,\left( \hat{\sigma}_{j}^{2}+\tau^{2} \right) \right),$$

where $\hat{\beta}_{j}$ is the G×E coefficient from study $j\in1,\ldots,8$; $\beta$ is the (assumed) true G×E effect; $\hat{\sigma}_{j}^{2}$ is the variance of the G×E coefficient as estimated from each study; and $\tau^{2}$ is the (assumed) between-study variance. The model assumes that the $\hat{\beta}_{j}$’s are a random sample from a Normal distribution with mean $\beta$ and variance $\left( \hat{\sigma}_{j}^{2}+\tau^{2} \right)$.

This model has two parameters: $\beta$ and $\tau^{2}$. To assess the power of the primary analysis in a variety of setting, we performed simulations for all possible combinations of $\beta=\left\{ 0.121,0.132,0.176,0.219,0.263,0.307,0.351,0.395,0.439,0.483,0.527 \right\}$ and $\tau^{2}=\left\{ 0,0.103 \right\}$.

$\beta=0.121$ is the point estimate from the random effects meta-analysis, adjusted model (2) (Table 1). $\beta=0.263$ is the point estimate reported by Steer et al. [[1](#_ENREF_1)], which we used in the sample size calculations in the meta-analysis protocol [[2](#_ENREF_2)], converted to standard deviation units. $\beta=0.132$ and $\beta=0.527$ are half of and twice the Steer et al.’s point estimate, which were also used in the protocol. The remaining estimates were chosen to form an equally-spaced sequence of 10 values, starting at 0.132 and ending at 0.527 (and thus centred at 0.263).

Setting $\tau^{2}=0$ implies assuming no between-study heterogeneity. $\tau^{2}=0.103$ is simply using the between-study variance estimated in our meta-analysis.

Finally, we also compared the power of fixed and random effects meta-analysis.

For each scenario, 10,000 summary datasets were simulated. Power was calculated as the proportion of datasets where *P*<0.05.

# Study Acknowledgements

**1982 Pelotas Birth Cohort Study**

The 1982 Pelotas Birth Cohort Study is conducted by the Postgraduate Program in Epidemiology at Federal University of Pelotas (*Universidade Federal de Pelotas*) in collaboration with the Brazilian Public Health Association (ABRASCO). From 2004 to 2013, the Wellcome Trust supported the study. The International Development Research Center, World Health Organization, Overseas Development Administration, European Union, National Support Program for Centers of Excellence (PRONEX), the Brazilian National Research Council (CNPq), and the Brazilian Ministry of Health supported previous phases of the study.

Genotyping was supported by the Department of Science and Technology (DECIT, Ministry of Health) and National Fund for Scientific and Technological Development (FNDCT, Ministry of Science and Technology), Funding of Studies and Projects (FINEP, Ministry of Science and Technology, Brazil), Coordination of Improvement of Higher Education Personnel (CAPES, Ministry of Education, Brazil).

More information about the 1982 Pelotas Birth Cohort Study are available in cohort profile papers by Victora and Barros (PMID: 16373375), and by Horta et al. (PMID: 25733577).

**Avon Longitudinal Study of Parents and Children (ALSPAC)**

We are extremely grateful to all the families who took part in this study, the midwives for their help in recruiting them, and the whole ALSPAC team, which includes interviewers, computer and laboratory technicians, clerical workers, research scientists, volunteers, managers, receptionists and nurses. The UK Medical Research Council and Wellcome (Grant ref: 102215/2/13/2) and the University of Bristol provide core support for ALSPAC. This publication is the work of the authors and Fernando Pires Hartwig will serve as guarantors for the contents of this paper. Genetic data was generated by Sample Logistics and Genotyping Facilities at Wellcome Sanger Institute and LabCorp (Laboratory Corportation of America) using support from 23andMe.
**Copenhagen Prospective Study on Asthma in Childhood (COPSAC) 2010**

We greatly acknowledge the private and public research funding allocated to COPSAC and listed on [www.copsac.com](http://www.copsac.com), with special thanks to The Lundbeck Foundation (Grant nr. R16-A1694); Ministry of Health (Grant nr. 903516); Danish Council for Strategic Research (Grant nr.: 0603-00280B); The Danish Council for Independent Research and The Capital Region Research Foundation as core supporters. The funding agencies did not have any influence on study design, data collection and analysis, decision to publish or preparation of the manuscript. No pharmaceutical company was involved in the study. We gratefully express our gratitude to the participants of the COPSAC 2010 study for all their support and commitment. We also acknowledge and appreciate the unique efforts of the COPSAC research team.

**Dunedin Multidisciplinary Health and Development Study**

The Dunedin Multidisciplinary Health and Development Research Unit is funded by the New Zealand Health Research Council and the New Zealand Ministry of Business, Innovation and Employment (MBIE). Research was supported by grants from the National Institute on Aging (AG032282), National Institute of Child Health and Development (HD077482), and Medical Research Council (MR/P005918/1). We thank the Dunedin Study founder Phil Silva. More information about the Dunedin Study is available in a cohort profile paper by Poulton, Moffitt, and Silva (PMID: 25835958).

**Generation R Study**

The Generation R Study is conducted by researchers at the Erasmus Medical Center in close collaboration with the School of Law and Faculty of Social Sciences of the Erasmus University Rotterdam; the Municipal Health Service for the Rotterdam area; the Rotterdam Homecare Foundation; and the Stichting Trombosedienst and Artsenlaboratorium Rotterdam. We gratefully acknowledge the contributions of general practitioners, hospitals, midwives and pharmacies in Rotterdam. The Generation R Study is made possible by financial support from the Erasmus Medical Center, Rotterdam, the Erasmus University Rotterdam, the Netherlands Organization for Health Research and Development (ZonMw), the Netherlands Organisation for Scientific Research (NWO), and the Ministry of Health, Welfare and Sport. H.T. received additional grants from the Netherlands Organization for Health Research and Development (ZonMw VIDI 017.106.370).

More information about The Generation R Study is available in cohort profile paper by Jaddoe et al. (PMID: 20967563).

**INMA (*INfancia y Medio Ambiente* – Environment and Childhood)**

Population-based birth cohorts were established as part of the *INfancia y Medio Ambiente* (INMA) Project in several regions of Spain following a common protocol. The present analysis uses the INMA subcohorts of Menorca, Valencia, Sabadell, and Gipuzkoa. More information about the INMA project is available in a cohort profile paper (PMID: 21471022), and in the INMA webpage (<http://www.proyectoinma.org/>).

This study was funded by grants from Instituto de Salud Carlos III [G03/176, CB06/02/0041, 97/0588, 00/0021-2, FIS PI041436, PI06/0867, PI061756, PI081151, PI041705, and PS09/00432, PS09/00090, PS0901958, FIS-FEDER 03/1615, 04/1509, 04/1112, 04/1931, 05/1079, 05/1052, 06/1213, 07/0314, 09/02647, 11/0178, 11/02591, 11/02038, 13/1944, 13/2032, 14/0891, and 14/1687, PI14/00677 incl. FEDER funds], Spanish Ministry of Science and Innovation [SAF2008-00357], European Commission [ENGAGE project and grant agreement HEALTH-F4-2007-201413, FP7-ENV-2011 cod 282957 and HEALTH.2010.2.4.5-1], CIBERESP, Fundació La Marató de TV3 (090430), Generalitat de Catalunya-CIRIT 1999SGR 00241, Beca de la IV convocatoria de Ayudas a la Investigación en Enfermedades Neurodegenerativas de La Caixa, and EC Contract No. QLK4-CT-2000-00263, Conselleria de Sanitat Generalitat Valenciana, Department of Health of the Basque Government (2005111093 and 2009111069) and the Provincial Government of Gipuzkoa (DFG06/004 and DFG08/001), and Fundación Roger Torné.

The authors would particularly like to thank all the participants for their generous collaboration. The authors are grateful to Silvia Fochs, Anna Sànchez, Maribel López, NuriaPey, Muriel Ferrer, AmparoQuiles, Sandra Pérez, Gemma León, Elena Romero, and Amparo Cases for their assistance in contacting the families and administering the questionnaires. A full roster of the INMA Project Investigators can be found at <http://www.proyectoinma.org/presentacion-inma/listado-investigadores/enlistado-investigadores.html>.

**Saguenay Youth Study**

We thank all families who took part in the Saguenay Youth Study and the following individuals for their contributions in designing the protocol, acquiring and analyzing the data: psychometricians (Chantale Belleau, Mélanie Drolet, Catherine Harvey, Stéphane Jean, Hélène Simard, Mélanie Tremblay, Patrick Vachon), ÉCOBES team (Nadine Arbour, Julie Auclair, Marie-Ève Blackburn, Marie-Ève Bouchard, Annie Gautier, Annie Houde, Catherine Lavoie), laboratory technicians (Denise Morin and Nadia Mior), nutritionists (Caroline Benoit and Henriette Langlais), MRI team (Sylvie Masson, Suzanne Castonguay, Marie-Josée Morin, Caroline Mérette), and cardio nurses (Jessica Blackburn, Mélanie Gagné, Jeannine Landry, Catherine Lavoie, Lisa Pageau, Réjean Savard, France Tremblay, Jacynthe Tremblay). We thank Dr. Jean Mathieu for the medical follow up of participants in who we detected any medically relevant abnormalities. We thank Manon Bernard for designing and managing our online database. We thank Dr. Jean Shin for her statistical advice.

The Saguenay Youth Study has been funded by the Canadian Institutes of Health Research (TP, ZP), Heart and Stroke Foundation of Canada (ZP), and the Canadian Foundation for Innovation (ZP). Computations were performed on the GPC supercomputer at the SciNet HPC Consortium. SciNet is funded by: the Canada Foundation for Innovation under the auspices of Compute Canada; the Government of Ontario; Ontario Research Fund - Research Excellence; and the University of Toronto.

***Småbørn Kost Og Trivsel* (SKOT)-I and SKOT-II**

We gratefully acknowledge the contribution of all the families and children who participate in the study. SKOT-I was funded by the Danish Directorate for Food, Fisheries and Agricultural Business as part of the project Complementary and Young Child Feeding (CYCF) – Impact on Short- and Long-Term Development and Health. SKOT-II was partially funded by grants from Aase and Ejnar Danielsens Foundation and Augustinus Foundation and further funding was provided by the research program “Governing Obesity” funded by the University of Copenhagen Excellence Program for Interdisciplinary Research (http: //www.go.ku.dk). The Novo Nordisk Foundation Center for Basic Metabolic Research is an independent research center at the University of Copenhagen partially funded by an unrestricted donation from the Novo Nordisk Foundation (www.metabol.ku.dk).

The SKOT-I and SKOT-II cohorts were initiated by Kim F. Michaelsen and Lotte Lauritzen initiated genotyping of *FADS2* polymorphisms in these studies. The actual genotyping was performed by Theresia M. Schnurr under supervision of Torben Hansen.

More information about the SKOT-I and SKOT-II cohorts are available in previously published papers from the cohorts (PMID: 21059086 and 25646329) and (PMID: 25469467 and 26111966), respectively.

**Western Australian Pregnancy Cohort (Raine) Study**

The authors are grateful to the Raine Study participants and their families, and to the Raine Study Team for cohort coordination and data collection. The authors gratefully acknowledge the NH&MRC for their long term contribution to funding the study over the last 25 years and also the following Institutions for providing funding for Core Management of the Raine Study: The University of Western Australia (UWA), Raine Medical Research Foundation, UWA Faculty of Medicine, Dentistry and Health Sciences, Telethon Kids Institute, Women and Infants Research Foundation, Curtin University and Edith Cowan University.

The authors gratefully acknowledge the assistance of the Western Australian DNA Bank (National Health and Medical Research Council of Australia National Enabling Facility). This study was supported by the National Health and Medical Research Council of Australia [grant numbers 572613 and 403981] and the Canadian Institutes of Health Research [grant number MOP-82893]. Nicole M. Warrington is supported by a National Health and Medical Research Council Early Career Fellowship (APP1104818).

This work was supported by resources provided by the Pawsey Supercomputing Centre with funding from the Australian Government and the Government of Western Australia.

# References

1. Steer CD, Davey Smith G, Emmett PM, Hibbeln JR, Golding J. FADS2 polymorphisms modify the effect of breastfeeding on child IQ. *PLoS One* 2010; 5(7):e11570.

2. Hartwig FP, Davies NM, Horta BL, Victora CG, Davey Smith G. Effect modification of FADS2 polymorphisms on the association between breastfeeding and intelligence: protocol for a collaborative meta-analysis. *BMJ Open* 2016; 6(6):e010067.
